# Microsatellite Loci Reveal High Genetic Diversity, Mutation, and Migration Rates as Invasion Drivers of Callery Pear (*Pyrus calleryana*) in the Southeastern United States

**DOI:** 10.3389/fgene.2022.861398

**Published:** 2022-04-05

**Authors:** Shiwani Sapkota, Sarah L. Boggess, Robert N. Trigiano, William E. Klingeman, Denita Hadziabdic, David R. Coyle, Marcin Nowicki

**Affiliations:** ^1^ Department of Entomology and Plant Pathology, University of Tennessee, Knoxville, TN, United States; ^2^ Department of Plant Sciences, University of Tennessee, Knoxville, TN, United States; ^3^ Department of Forestry and Environmental Conservation, Clemson University, Clemson, SC, United States

**Keywords:** “Bradford” pear, invasive species, genetic variability, ornamental trees, population structure

## Abstract

*Pyrus calleryana* Decne. (Callery pear) is a deciduous tree native to China, Japan, Korea, and Taiwan. It is a popular ornamental tree in the United States (US) with early spring blooms and vibrant fall color. There are at least 26 cultivars of *P. calleryana* available in the US of which “Bradford” is the most well-known. Open-pollinated *P. calleryana* escapees are becoming one of the most common invasive tree species in the eastern United States. Developing better management practices for invasive *P. calleryana* requires detailed knowledge about reproductive biology and genetic diversity of the species, however, little is currently known about genetic variability within those open-pollinated populations. We investigated genetic diversity and population structure of non-cultivated, escaped *P. calleryana* populations within a ∼177 km radius in the southeastern United States. Because *P. calleryana* exhibits a range of morphological variation with great evolutionary potential, we hypothesized that a high genetic diversity would be manifested among escaped *P. calleryana*. Using 15 previously developed microsatellite loci, we genotyped 180 open-pollinated *P. calleryana* individuals that were collected across six naturally occurring sites in Tennessee, Georgia, and South Carolina, United States. Our results demonstrated the presence of a population structure with high genetic diversity, high gene flow, and high genetic differentiation between individuals across collection sites. Our results revealed that *P. calleryana* populations had differentiated shortly after the introduction to the US, most likely from specimens imported from Asia, consistent with historical records and our prior findings. The high invasive potential of the species is perhaps best underscored by transformation of *P. calleryana* specimens introduced from Asia into escape populations at continental scale across the United States. Our data also provided novel insight into potential issues that could be problematic for the future as *P. calleryana* may pose a potential threat to the economy, ecology, and native biodiversity in invaded areas.

## Introduction

Invasive species cause great economic, social, and ecological threats to both natural and managed ecosystems (Poland et al., 2021) and are the second major cause of endangerment and extinction of native species (Crowl et al., 2008). Invasive species generated an estimated annual mean cost of $162.7 billion (USD) worldwide in 2017 (Diagne et al., 2021). Many plant species originally introduced for horticultural purposes have escaped cultivation and have imposed a high economic cost for their control (Reichard and White, 2001; Bell et al., 2003; Li et al., 2004). For example, the annual control cost of invasive Chinese and European privet species in the US averages up to $527 million (Benez-Secanho et al., 2018). Estimates of cost impacts generated by plants invasive to the US amount to $190.5 billion for the period between 1960 and 2020 (Fantle-Lepczyk et al., 2022).

A successful plant invasion includes an introduction, establishment of the species in an unintended area, and a lag phase followed by colonization of additional areas (Sakai et al., 2001). A plant species could be introduced in a new area either through natural spread (e.g., seeds or pollen hybridizing with compatible species), accidentally (e.g., transportation or inadvertent human introduction), or deliberately (e.g., breeding/horticultural uses) (Culley and Hardiman, 2007). Although not all introduced plant species become invasive, those with the potential to do so possess certain traits including a high reproductive rate, ability to thrive in adverse environmental conditions, and rapid growth (Thompson, 1991; Holzmueller and Jose, 2009).

*Pyrus calleryana* Decne. (Callery pear) is native to China, Taiwan, Korea, and Japan (Rubtsov, 1944; Bell and Zimmerman, 1990; Cuizhi and Spongberg, 2003). Introduced for horticultural purposes, it has subsequently become invasive in Australia and the United States (Csurhes and Edwards, 1998; Barker et al., 2006; Culley and Hardiman, 2007). Among many cultivars that have been commercialized for landscape use, ‘Bradford’ is considered the most widely planted and commonly known ornamental cultivar of *P*. *calleryana* in the United States *Pyrus calleryana* cultivars are used as deciduous landscape trees prized for their shade, bright white flowers, and colorful fall foliage. Young plants begin to produce small flowers as early as 3 years of age and the flowers are generally described as having an unpleasant odor (Bell and Zimmerman, 1990; Cuizhi and Spongberg, 2003). Fruits serve as an emergency food source for birds and other vertebrates; thus, *P*. *calleryana* seed dispersal by animals likely contributes to the plant’s spread.

In the early 1900s, *P*. *calleryana* accessions were imported to the US to introduce resistance to fire blight causing bacteria, *Erwinia amylovora* Burrill, into *P*. *communis* L. Several *P*. *calleryana* selections exhibited desirable horticultural traits for urban use, which led to the development and release of many hybrid cultivars including other *Pyrus* species (Culley and Hardiman, 2007). By 1962, ‘Bradford’ was available commercially in the US, yet instances of intraspecific hybridization were becoming evident, as were hybridization events between other released cultivars (Whitehouse et al., 1963; USDA, 1981; Culley and Hardiman, 2009; Dunn, 2018). Rootstocks of *P*. *calleryana* trees, which are used for grafting with other species, are routinely produced either from seeds or *via* vegetative propagation. As a result, the scion and the rootstock of a given commercial individual represent two different genotypes. The shoot sprouts of such rootstock can potentially flower resulting in cross-pollination with the scion of the same individual. In addition, the pollen may be dispersed short distances via various pollinators and the seeds across longer distances via migratory birds, mammals, and human activities (Vincent, 2005; Culley et al., 2011). Cultivars of *P*. *calleryana* were not expected to escape cultivation as the species is self-incompatible, propagated by vegetative methods, and produces very few seeded fruits in the native range (Gilman and Watson, 1994; Culley and Hardiman, 2007). Furthermore, *P*. *calleryana* trees in the native range often grow quite small, and are widely scattered in China (Culley and Hardiman, 2007). However, soon after the release of commercial ornamental cultivars in the United States, the species was observed in several natural habitats with the first reported escapes identified in 1964 in eastern Arkansas (Vincent, 2005). Since then, the escaped *P*. *calleryana* trees were increasingly found in the natural areas of the Eastern United States (Swearingen et al., 2002; Vincent, 2005). ‘Bradford’ and related cultivars were recognized for their invasive potential in 1994, and approximately 10 years later the naturalized, non-cultivated *P*. *calleryana* trees were found in at least 26 states (Vincent, 2005). Currently, invasive *P*. *calleryana* are reported to be distributed in at least 33 US states (EDDMapS, 2021). The species is predicted to have the potential of becoming one of the most problematic invasive plants in the United States (Culley et al., 2011; Warrix and Marshall, 2018; Coyle et al., 2021; Sapkota et al., 2021).

Despite the wide prevalence of *P*. *calleryana* in the United States, we have limited knowledge about its biology, ecology, and spatial distribution. Assessment of plant genetic diversity using molecular data helps us understand the adaptation dynamics and spread characteristics (Purugganan, 2000). For invasive species, a fine-scale study across a narrow geographic area may help infer the dynamics of natural dispersal through pollen, seed, or root sprouts of unknown genetic composition (Darling and Folino-Rorem, 2009). The fine-scale distribution patterns of the invasive populations, even with low genetic structure, may help reveal important evolutionary patterns (Short and Petren, 2011).

Invasive plant management and control strategies should be developed based on genetic and biological characteristics (Allendorf and Lundquist, 2003). In species deemed highly invasive, great variability in their genetic diversity, gene flow patterns, resistance/tolerance to control methods, and modes of reproduction are expected, collectively requiring targeted management practices for each species (Gaskin et al., 2014). For example, locally-effective management practices for *P*. *calleryana* currently only include the complete removal of trees (Swearingen et al., 2002; Culley and Hardiman, 2007) or herbicide applications (Vogt et al., 2020). However, this only may be feasible across smaller areas such as urban environments or local plantations. Although prescribed burning is comparatively more cost-effective, this treatment results in increased resprouting of *P. calleryana* (Warrix and Marshall, 2018). We lack effective and economically feasible management options for *P*. *calleryana,* and available measures currently include general rather than targeted management practices. Hence, it is imperative to understand the biology of the species so that effective targeted management strategies can be formulated accordingly.

Most research studies in *Pyrus* spp. are limited to the identification and characterization of cultivars/species using various DNA markers (Iketani et al., 1998; Yamamoto et al., 2001; Yamamoto et al., 2002a; Yamamoto et al., 2002b; Yuanwen Teng et al., 2001; Bao et al., 2007; Bao et al., 2008). A few *P. calleryana* population studies have been conducted within the species native range (Liu et al., 2012; Kato et al., 2013; Sapkota et al., 2021), however, only limited information on non-cultivated populations in the US is available (Culley and Hardiman, 2009). Therefore, our goal was to assess the genetic diversity and population structure of invasive *P*. *calleryana* populations within a narrow geographic range using microsatellite loci. Because high genetic diversity was maintained among *P*. *calleryana* trees in their native range (Sapkota et al., 2021), we hypothesized there would be high genetic diversity among *P*. *calleryana* trees that escaped cultivation. To investigate this hypothesis, previously developed microsatellite loci (Sapkota et al., 2021) were used to address the following specific objectives: to 1) evaluate the fine-scale genetic diversity present within escaped *P*. *calleryana* populations within a ∼177 km radius area of Tennessee, Georgia, and South Carolina, United States; 2) investigate fine-scale patterns in spatial distribution and gene flow within *P*. *calleryana* trees in these locations; and 3) infer the evolutionary history of *P*. *calleryana* in relation to samples from the species native area and the US-developed cultivars as a source of origin, using Approximate Bayesian Computation.

## Materials and Methods

### Sample Collection and Genomic DNA Extraction

**FIGURE 1:**
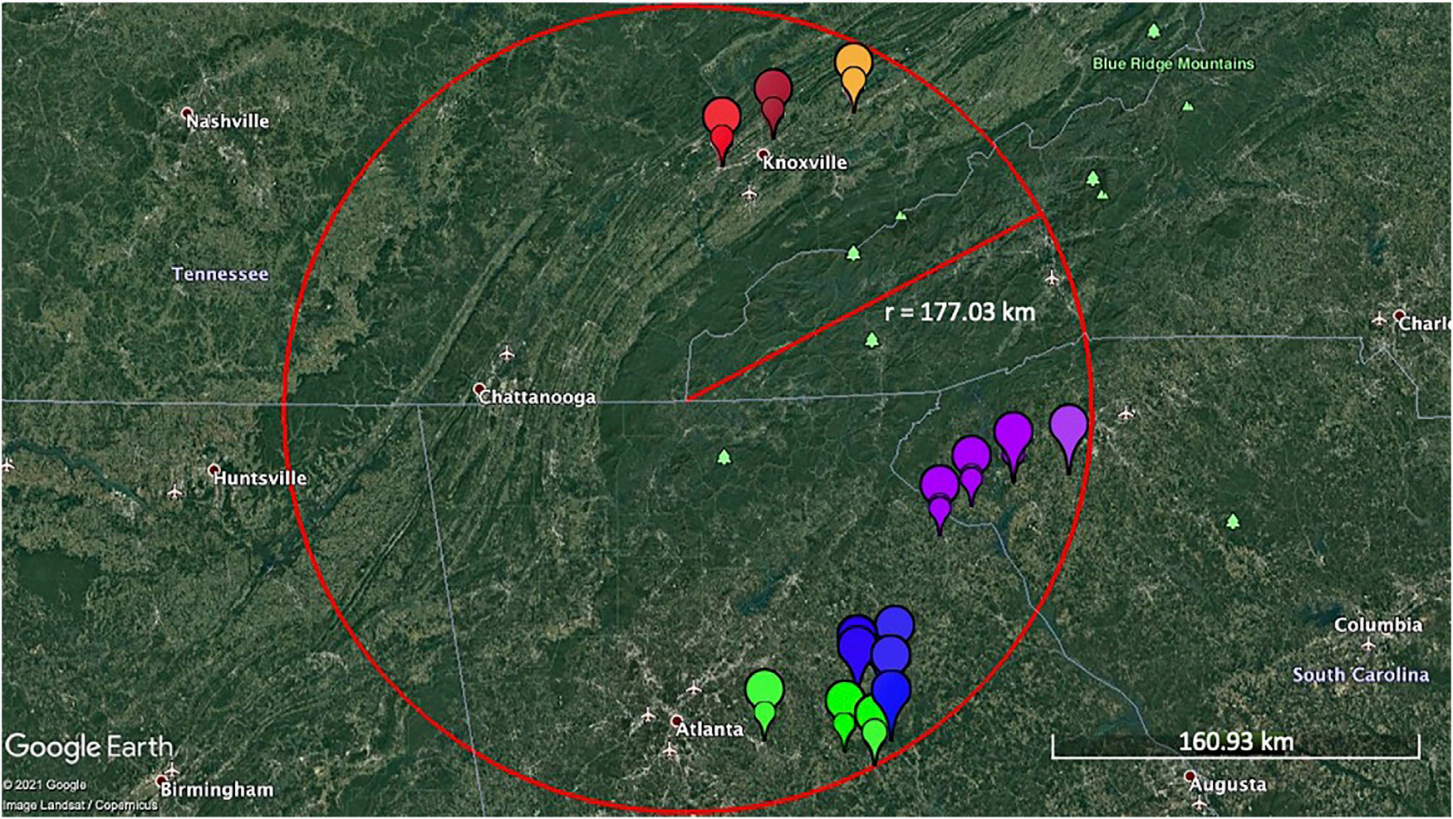
Collection sites of the open-pollinated *Pyrus calleryana* trees (*n* = 180) samples within a ∼177 km radius. Each colored symbol represents individual samples taken from different trees. Leaf samples from trees were collected from Tennessee, Georgia, and South Carolina, United States. Each of the six colors represent six subpopulations (Brown: North Group A, Red: North Group B, Orange: North Group C, Blue: South Group A, Green: South Group B, and Purple: South Group C). The scale indicates the ground-level distance of ∼161 km. The map was generated using Google Earth Pro version 7.3.

*Pyrus calleryana* leaf samples were collected from individual trees in Tennessee, Georgia, and South Carolina, United States (Figure 1). Six to eight healthy, fully expanded leaves were collected from each of the 30 wild/non-cultivated trees per collection site (considered a subpopulation for this study). Samples from a total of 180 trees were collected, of which 90 were from eastern Tennessee, and 90 from northeastern Georgia/northwestern South Carolina. Based on the site of sample collection, samples were grouped into “North Group” (*n* = 90) and “South Group” (*n* = 90). Both “North Group” and “South Group” contained three subpopulations of 30 individuals each, including North Group A; North Group B; North Group C, and South Group A; South Group B; South Group C, respectively (Figure 1; Supplementary Table S1). For one of the analyses described below (DIYABC), the genotyping information of samples from Sapkota et al. (2021) was used to gain access to the genotyping data from the originating materials from China, Japan, Korea, and the US-developed cultivars (*n* = 75); the remaining analyses used data generated in this study subdivided into two major groups or six subpopulations.

Each sample of approximately 100 mg of air-dried leaves (per individual tree) was homogenized using a Bead mill 24 (Fisher Scientific, Pittsburgh, PA, United States) and subjected to genomic DNA (gDNA) extraction using EZNA DNA DS Mini Kit (Omega Bio-Tek, Norcross, GA, United States), according to the manufacturer’s protocol. Nanodrop (Thermo Fisher Scientific, Wilmington, DE, United States) was used to measure the concentration and purity of the extracted gDNA samples.

### Microsatellite Primers and Genotyping Conditions

Genomic Short Sequence Repeats (gSSRs) for *P*. *calleryana* were developed (Sapkota et al., 2021) using genome sequence data of a closely related pear, *Pyrus × bretschneideri* Rehder (GenBank number: JH994112; Wu et al., 2013), because a high-quality genome sequence of *P*. *calleryana* was not available at the time. Considering high amplification rates, polymorphic character, and agreement with the expected PCR product size, 15 robust gSSRs were selected from Sapkota et al. (2021) and used in this study.

The gSSR loci were amplified using polymerase chain reaction (PCR) in a 10 μl reaction mixture consisting of the following: 1 μl of 4 ng gDNA, 5 μl of 2 × GoTaq^®^ DNA Polymerase (Promega, Madison, WI, United States), 1 μM final concentration of each primer, and 0.5 μl of dimethyl sulfoxide (DMSO). For the data validation, gDNA from *P*. *calleryana* var. *dimorphophylla* (collected from Japan in 1933 and maintained by Morton Arboretum, Chicago, IL, United States) was used as a positive control, and sterile water was used as a non-template control for each primer pair. A touch-down protocol was used for PCR amplification to increase the specificity of the amplified products (Korbie and Mattick, 2008). The following thermal profile was used for PCR amplification: initial denaturation at 94°C for 3 min, followed by 10 cycles of denaturation at 94°C for 30 s, annealing at 65°C for 30 s with a touchdown of 0.7°C/cycle and an extension at 72°C for 30 s then, followed by 30 cycles of denaturation at 94°C for 30 s, annealing at 58°C for 30 s and an extension at 72°C for 30 s, with a final extension of 72°C for 4 min.

Amplified PCR products were visualized using the QIAxcel Advanced Capillary Electrophoresis System (Qiagen, Germantown, MD, United States) and analyzed with a 15/600 bp alignment marker and 25 to 500 bp DNA size marker (Qiagen). Samples that failed to amplify after two subsequent PCR attempts were designated as missing data. Samples that failed to amplify in more than 5 loci were excluded from the study. Consequently, four samples failed to amplify and subsequent analyses included *P*. *calleryana* genotyped dataset of 176 tree samples within the six subpopulations.

### Data Analysis

The MS Excel macro FlexiBin (Amos et al., 2007) was used to transform the raw allelic sizes into the statistically identical allelic classes using the information of nucleotide repeat motif size. The binned allelic dataset was used for further data analyses. PGDSpider version 2.1.1.5 (Lischer and Excoffier, 2012) was used to transform the binned PCR allele sizes into repeat numbers. Clone correction of the dataset was performed using *poppr* version 2.8.5 (Kamvar et al., 2014) in RStudio version 1.2.5033 using R version 3.6.2 (R Core Team, 2013). No clonal multi-locus genotypes (MLGs) were found in both dataset subdivisions at population level (North/South Groups or six subpopulations), the genotyping data for those 176 unique samples were used for subsequent analyses.

### Population Genetics of *Pyrus calleryana*

#### Genetic Diversity

For each of the 15 gSSRs, the following genetic diversity indices were calculated in R using the packages *poppr* and *hierfstat* version 0.04-22 (Goudet, 2005): number of alleles detected (N), number of effective alleles (NAe; number of equally frequent alleles achieving the same H_e_ as observed in our study), rarefied allelic richness (A_r_), observed heterozygosity (H_o_), Nei’s unbiased expected heterozygosity (H_e_; Nei, 1978), and Jost’s differentiation estimate (D_est_; Jost, 2008). GenAlEx version 6.5 (Peakall and Smouse, 2006) was used to estimate the gene flow (N_m_ = ¼×[(1/F_ST_)-1]) and the presence of private alleles (P_a_) in the subpopulations. SPAGeDi (Hardy and Vekemans, 2015) was used to calculate the hierarchical fixation indices including inbreeding coefficient (F_IS_), allele fixation index (F_ST_), and their respective repeat number analogues estimated using the allele size and the motif length (R_IS_ and R_ST_) (Slatkin, 1995; Hardy and Vekemans, 2002). Analyses were performed independently for North/South Groups and the six subpopulations dataset subdivisions. To determine the significance of the hierarchical indices, 10,000 permutations were performed among gene copies in SPAGeDi (Pons and Petit, 1996).

Analysis of Molecular Variance (AMOVA) was performed for North/South Groups and six subpopulation dataset subdivisions, to estimate the molecular variance distribution among and within subpopulations using the package *poppr* with 1,000 permutations. Linkage Disequilibrium (LD) of the 15 gSSRs was assessed in *poppr* using 1,000 permutations. The pairwise index of association ( $\bar{r}$ _d_) was used for assessing the linkage among loci to identify a possible bias of the patterns of LD due to a single or few pair(s) of loci.

### Population Structure

#### Mantel Test for Isolation by Distance

The Mantel test was performed to estimate isolation by distance using package MASS version 7.3-50 (Ripley et al., 2013) with 1,000 permutations. Mantel test results were used to determine the correlation between genetic and geographical distance matrices of the individuals. The underlying correlative relationship between genetic and geographical distance matrices was confirmed with the Mantel correlogram test using packages *ade4* version 1.7-13 (Dray and Dufour, 2007) and *vegan* version 2.5-3 (Oksanen et al., 2013), at *α* = 0.05.

SPAGeDi was also used to investigate the contribution of mutation rate to the population structure of *P*. *calleryana* dataset using 10,000 permutations among alleles within each locus. Additionally, we used SPAGeDi to determine the phylogeographic patterns in *P*. *calleryana* dataset using 10,000 permutations of gene copies among individuals within populations or individuals among all populations.

#### STRUCTURE and Discriminant Analysis of Principal Components

The population structure of the *P*. *calleryana* dataset was analyzed using Bayesian approach in Structure version 2.3.4 (Pritchard et al., 2000). Thirty independent Monte Carlo Markov Chains (MCMC) were used with 250,000 generations of burn-in period and 750,000 run steps for each number of clusters (K = 1 to 10). PopHelper version 1.0.10 (Francis, 2017) with the Evanno method (Evanno et al., 2005) was then used to analyze and visualize the Structure results. ObStruct version 1.0 (Gayevskiy et al., 2014) was used to determine the correlation between the population structure of Structure-inferred ancestral profiles and the predefined subpopulations. This program uses an ad-hoc R^2^ statistic whose value ranges from 0 (admixture between populations/recent divergence) to 1 (population structure/complete divergence). Changes in the R^2^ statistic when the predefined/inferred populations are sequentially removed help infer the contributions of the predefined/inferred populations to the structure of the *P. calleryana* dataset.

A model-free multivariate clustering approach, Discriminant Analysis of Principal Components (DAPC), was also used to investigate the genetic structure of the *P. calleryana* dataset using the package *adegenet* version 2.1.1 (Jombart, 2008) in R. A principal component analysis (PCA) was performed and the PCA vectors explaining the majority of variance but minimizing the over-fit of DAPC were selected. The number of PCAs selected in this manner was then used to optimize, cross-check, and re-plot the DAPC analysis using 100 permutations across the increasing number of PCAs used. The result was confirmed using a dendrogram of the unrooted neighbor-joining tree of pairwise genetic distances among the six populations (Nei, 1978) using *ape* version 5.5 (Paradis et al., 2004) in R.

### Population Demography

#### Bottleneck

Bottleneck version 1.2.02 (Cornuet and Luikart, 1996) was used to investigate the evidence for an evolutionary recent bottleneck of *P*. *calleryana* populations for North and South Groups. Bottleneck outputs a graph as either an L-shaped graph indicating a stable population or a mode-shift graph indicating a population that experienced a bottleneck. We used a stepwise-mutation model (S.M.M.) and two-phase mutational model (T.P.M.) to test for a recent bottleneck or expansion of the *P*. *calleryana* populations. The variance of geometric distribution for T.P.M. was set to 12 along with 95% of S.M.M. Significance of the test under either of these models was evaluated using sign test, standardized differences test, and Wilcoxon sign-rank test with 10,000 simulations. The heterozygosity excess and heterozygosity deficiency of each group was assessed using Wilcoxon sign-rank test, whereas the results of all three basic tests i.e., sign, standardized differences, and Wilcoxon sign rank test, were used to assess the mode-shift in population size.

#### Do-It-Yourself Approximate Bayesian Computation

The population history of *P*. *calleryana* was investigated with the Approximate Bayesian Computation approach using the program DIYABC version 2.1 (Cornuet et al., 2014). For this analysis only, *P*. *calleryana* samples from the species native area and the US-developed cultivars were used as the originating population (Sapkota et al., 2021; *n* = 75; designated hereafter as “Origin Group”). Based on the geographical location and support by both Structure and DAPC analysis, the collected samples were divided into 3 major populations, i.e., “North Group,” “South Group,” and “Origin Group”.

To assess the parameter values for the main DIYABC run, an initial run that used the entire dataset as one population was computed with the following parameters under uniform distribution: population size (min: 10; max: 10000); time (min: 10; max: 10000), and a generalized S.M.M. with a mean mutation rate of 5 × 10^–4^ (min: 10^–5^; max: 10^–2^) mutations per generation per locus. To explore this parameter space, one million pseudo-observed datasets (PODs) were simulated, and based on the 95% confidence intervals of the results (95% CI) the parameter values for the main runs were established.

In the main run, the genotyped dataset was tested using 6 possible evolutionary scenarios devised from the history of *P. calleryana* introduction to the United States (Whitehouse et al., 1963; Culley, 2017). Those scenarios considered population divergence, admixture, and the presence of an unsampled intermediary population (“Ghost” population; Cornuet et al., 2015). Scenario 1 assumed concurrent divergence of North Group and South Group from the Origin Group. Scenario 2 included an unsampled intermediary population diverging from Origin Group, and later giving rise to North Group and South Group. Scenario 3 assumed sequential independent divergence events from the Origin Group—first to the North Group, then to the South Group. Scenario 4 was similar to Scenario 3, but with reversed order of divergence. Scenario 5 considered divergence of North Group from Origin Group, and at a later time, divergence of South Group from North Group. Scenario 6 assumed divergence of South Group from Origin Group, and at a later time, divergence of North Group from South Group. For each scenario, 1 million PODs were simulated to explore the parameter space under uniform distribution: population size (min: 100; max: 100000) and time of splits in generations (min: 1; max: 10000 with t2 ≥ t1). We assumed a uniform prior distribution and a generalized S.M.M. with a mean mutation rate of 5.00 × 10^–4^ (ranging from 5.44 × 10^–4^ to 8.00 × 10^–2^ mutations per generation per locus), as per the 95% CI from the initial run. The following summary statistics for each scenario were calculated by the DIYABC program: the mean number of alleles, mean genetic diversity, mean population size variance, classification index, pairwise F_ST_, and distance between pairs of populations (dμ)^2^. The effective population sizes for each population created within the DIYABC analysis, expressed as median of number of individuals, are mathematically estimated constructs and should not be treated as exact values; rather, the relative comparisons of population sizes provide information of the species demographic changes in time. For each scenario, summary statistics of the PODs were compared with that of the observed dataset of genotyped *P*. *calleryana* trees.

The logistic regression analysis on 1% of the PODs closest to the observed dataset was used to infer the relative posterior probabilities of the scenarios undergoing comparisons (Cornuet et al., 2010). Two scenarios having the highest posterior probabilities from the main DIYABC run were used for subsequent comparative analyses. The bias and precision analysis on parameter estimations was computed on the dataset for the best-supported scenario. For these two best-supported scenarios, the “confidence in scenario choice” program option was used to test the goodness of fit by estimating the type I and type II errors and prior/posterior errors based on 1,000 PODs.

## Results

### Population Genetics of *Pyrus calleryana*

#### Genetic Diversity

All 176 *P*. *calleryana* individuals in North/South Groups and six subpopulations represented unique multi-locus genotypes (MLGs) and were used for further downstream analyses. Overall, 2.8% of missing data were detected across the entire dataset (Tables 1, 2). The locus PyC032 had the highest missing data of 10.2% and the subpopulation South Group B had 5.3% of missing data (Tables 1, 2). Neither North Group nor South Group followed the Hardy-Weinberg equilibrium (HWE) (Supplementary Figure S1). The gSSRs chosen for the study were powerful in discriminating MLGs as only 6 gSSRs were necessary to detect all the MLGs present in the dataset (Supplementary Figure S2).

**TABLE 1:** Genetic diversity indices of trees genotyped in the *Pyrus calleryana* dataset for six subpopulations and North/South Groups using fifteen microsatellite loci.

Subpopulations	N^c^	% Missing	# Alleles	NAe	$\bar{r}$ _d_	H_o_	H_e_	A_r_	F_IS_	P_a_
North Group A	30	0.20	8	5	0.01***	0.41	0.74****	8.04	0.45****	2
North Group B	30	0.40	7	4	0.03***	0.35	0.69****	6.99	0.50****	0
North Group C	30	3.30	6	3	0.04***	0.27	0.63****	5.50	0.58****	0
South Group A	29	2.30	7	4	0.06***	0.29	0.68****	6.75	0.57****	0
South Group B	29	5.30	7	4	0.02***	0.32	0.66****	6.32	0.52****	0
South Group C	28	5.20	8	5	0.01***	0.31	0.71****	7.34	0.57****	0
Summary statistics	176^a^	2.80^b^	12^b^	4^b^	0.03^b^***	0.32^b^	0.74^b^****	8.24^b^	0.56^b^****	2^a^
**Groups**	**N**	**% Missing**	**# Alleles**	**NAe**	$\bar{r}$ _**d**_	**H** **_o_**	**H_e_**	**A_r_**	**F_IS_**	**P_a_**
North Group	90	1.30	10	4	0.03***	0.34	0.71****	9.86	0.52****	2
South Group	86	4.30	9	4	0.03***	0.31	0.69****	8.93	0.56****	0
Summary statistics	176^a^	2.80^b^	12^b^	5^b^	0.03^b^***	0.32^b^	0.74^b^****	10.43^b^	0.56^b^****	2^a^

^a^: Summation (Σ); ^b^: Overall; ^c^N: number of samples used for the study in each population group; % missing: % of data missing in the given population group; # Alleles: Number of alleles detected; NAe: Effective number of alleles; $\;\underline{r}$ _d_: Standardized index of association considering the number of loci sampled (Kamvar et al., 2014); H_e_: Nei’s gene diversity corrected for sample size (Nei, 1978); H_o_: Observed heterozygosity; A_r_: Allelic richness; F_IS_: individual inbreeding coefficient; P_a_: Number of private alleles in each population. Significance of the dataset was assessed by 10,000 permutations and coded as follows: *p* < 0.0001 = ****; *p* < 0.001 = ***; *p* < 0.01 = **; *p* < 0.05 = *; *p* > 0.05 = ^ns^.

**TABLE 2:** Genetic diversity indices for six subpopulations of trees genotyped in the *Pyrus calleryana* dataset based on molecular data using fifteen microsatellite loci.

SSR locus	# Alleles^c^	% Missing	H_o_	H_e_	R_ST_	R_IS_	D_est_	N_m_
PyC006	13	8.50	0.06	0.81****	0.31****	0^ns^	0.57	0.86
PyC008	15	0.60	0.45	0.82****	0.38****	0.29*	0.52	1.14
PyC013	10	0.00	0.49	0.70*	-0.01^ns^	0.10^ns^	0.04	6.99
PyC014	16	0.60	0.25	0.86***	0.07**	0.67****	0.18	4.68
PyC015	13	3.40	0.24	0.84****	0.04ns	0.84****	0.37	2.11
PyC017	19	5.10	0.54	0.90^ns^	0.03*	0.18*	0.09	8.16
PyC018	13	1.70	0.48	0.84^ns^	0.03*	0.05^ns^	0.06	8.08
PyC020	14	0.00	0.34	0.76****	0^ns^	0.27**	0.16	3.33
PyC031	13	5.70	0.58	0.78****	0.02^ns^	-0.25^ns^	0.28	2.17
PyC032	8	10.20	0.03	0.55**	0.04*	0.83****	0.06	3.29
PyC035	12	0.60	0.53	0.86****	0.05**	0.04^ns^	0.19	4.80
PyC041	9	0.60	0.46	0.70**	-0.01^ns^	0.55****	0.07	5.07
PyC042	10	0.60	0.06	0.40*	0.08***	0.61****	0.11	2.45
PyC047	12	2.80	0.28	0.84****	0.27****	0.77****	0.47	1.53
PyC050	8	1.10	0.09	0.42^ns^	0.14****	0.32**	0.03	4.41
Summary statistics	185^a^	2.80^b^	0.32^b^	0.74^b^****	0.08^b^****	0.31^b^****	0.21^b^	3.94^b^

^a^Summation (Σ); ^b^: Overall; ^c^# Alleles: Number of alleles identified; H_o_: Observed heterozygosity; H_e_: Expected heterozygosity (Nei’s unbiased gene diversity; Nei, 1978); R_ST_, and R_IS_, are complementary measures of F_ST_ (fixation index) and F_IS_ (inbreeding coefficient) respectively; D_est_: Jost’s differentiation estimate (Jost, 2008); Nm: Gene flow given as N_m_ = ¼×[(1/F_ST_)-1]. Significance of the dataset was assessed by 10,000 permutations and coded as follows: *p* < 0.0001 = ****; *p* < 0.001 = ***; *p* < 0.01 = **; *p* < 0.05 = *; *p* > 0.05 = ^ns^.

Twelve alleles per population and 4 or 5 effective alleles per population were identified, depending on the data subdivision (Table 1). The overall observed heterozygosity (H_o_ = 0.32) was lower than the overall expected heterozygosity (H_e_ = 0.74) indicating the presence of high genetic diversity and of population structure (Table 1). The only private alleles detected were in the North Group (Table 1). High overall allelic richness (A_r_) detected in both dataset subdivisions suggested the long-term adaptability potential of *P*. *calleryana* individuals in the sampled region. In contrast, a positive inbreeding coefficient (F_IS_ = 0.56, *p* < 0.001) suggested that a substantial level of homozygosity observed within the dataset likely resulted from inbreeding (Table 1). Congruently, the standardized index of association ( $\bar{r}$ _d_) was 0.03 (*p* < 0.001) (Table 1). The values of pairwise LD ( $\bar{r}$ _d_) ranged from 0.01 to 0.06 (*p* = 0.002), which indicated the absence of linked loci and the genome-wide distribution of the gSSRs (Supplementary Figure S3).

In the locus-wise manner, an average of about 12 alleles per locus (ranging from 8 to 19) were detected across the dataset (Table 2). Our data suggested a high overall genetic differentiation (D_est_ = 0.21), indicating the presence of population structure in the tested *P*. *calleryana* dataset. Our data also indicated the presence of high gene flow (N_m_ = 3.94).

AMOVA was used to assess the proportion of molecular variance partitioned within the *P*. *calleryana* dataset for both dataset subdivisions, as well as the inclusion of variance within the individuals. In the 3-tier AMOVA, for the six subpopulation data sets, a low proportion of molecular variance was present among populations (6.80%) and the major portions of molecular variance were attributed to within individuals (43.38%) and within populations (49.82%) (Table 3). Comparably, differences in the North/South Groups dataset subdivision were negligible, with a small increase among populations (7.79%). When the within-individuals variance tier was excluded (2-tier AMOVA), the most of variance was attributed to within-populations tier; the six subpopulations dataset reached 90.77%, whereas the two groups dataset reached 89.28% (Table 3). This variation partitioning implies stable levels of inter-population variance, and intense intraspecific hybridization due to gene flow. The significant result of these tests (*p* < 0.001) demonstrated the existence of population structure within the *P*. *calleryana* dataset regardless of its subdivision.

**TABLE 3:** AMOVA of trees genotyped in the *Pyrus calleryana* dataset using six subpopulations and North/South Groups. AMOVA was performed at 3-tiers and 2-tiers without “Variations within individuals” in independent analyses.

3-Tiers AMOVA						
Six Subpopulations						
Source of variation	df^a^	Sum of squares	Mean squares	Sigma	% Variance	**Φ**
Variations among populations	5	229.64	45.93	0.58**	6.80	0.57
Variations within populations	170	2060.25	12.12	4.22**	49.82	0.53
Variations within individuals	176	646.93	3.68	3.68**	43.38	0.06
Total Variations	351	2936.82	8.37	8.37**	100.00	
**North and South Groups**						
**Source of Variation**	**df**	**Sum of Squares**	**Mean Squares**	**Sigma**	**% Variance**	**Φ**
Variations among populations	1	131.89	131.89	0.68**	7.79	0.58
Variations within populations	174	2158	12.40	4.36**	50.05	0.54
Variations within individuals	176	646.93	3.68	3.68**	42.16	0.08
Total Variations	351	2936.82	8.37	8.72**	100.00	
**2-Tiers AMOVA**						
**Six Subpopulations**						
**Source of variation**	**df** ^a^	**Sum of squares**	**Mean squares**	**Sigma**	**% Variance**	**Φ**
Variations among populations	5	168.18	33.64	0.86**	9.23	
Variations within populations	170	1435.35	8.44	8.44**	90.77	
Total Variations	175	1603.53	9.16	9.30**	100.00	0.09
**North and South Groups**						
**Source of Variation**	**df**	**Sum of Squares**	**Mean Squares**	**Sigma**	**% Variance**	**Φ**
Variations among populations	1	99.88	99.88	1.04**	10.72	
Variations within populations	174	1503.64	8.64	8.64**	89.28	
Total Variations	175	1603.53	9.16	9.68**	100.00	0.11

^a^df: Degree of freedom (sample size–1); Sum of Squares: Sum of squares of deviations of the observations from mean; Mean Squares: Sample variance as given by the sum of squares divided by the respective df; Sigma: Variance given for each hierarchical level; % Variance: Total variance percent for each hierarchical level; Φ: Statistics given by the test; Significance of the test was assessed using 1,000 permutations; ** = *p* < 0.001.

### Population Structure

#### Mantel Test for Isolation by Distance

**FIGURE 2:**
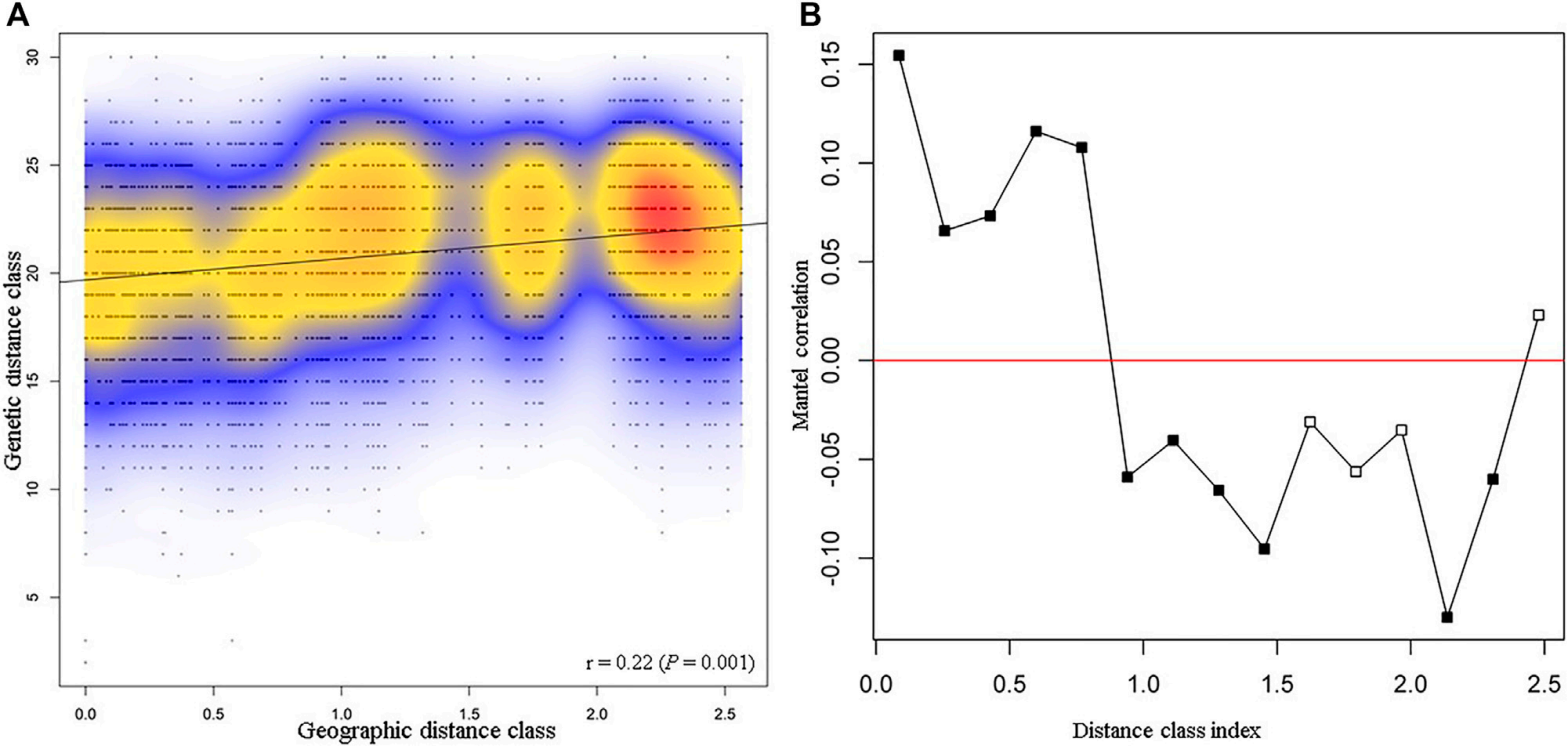
Mantel test results from the *Pyrus calleryana* dataset. Mantel test **(A)** and Mantel isolation-by-distance correlogram **(B)** for samples included within the *P. calleryana* dataset using 1,000 permutations. Distance class index (in 100 s of km) represents the maximum linear distance between samples, i.e., 260 km. Correlograms in **(B)** marked with solid black symbols are significant at α = 0.05.

Isolation by distance using the Mantel test was used to determine the correlation between genetic and geographic distances among *P*. *calleryana* individuals (Figure 2). We found a positive correlation between genetic and geographic distances (Mantel’s r = 0. 22, *p* = 0.001), indicating that about *r* ^2^ = 4.71% of the genetic variance observed can be explained by the geographic distance assessed. The maximum linear distance between samples was approximately 260 km. Across space, there was a non-linear relationship between genetic and geographic distances of *P*. *calleryana* individuals, indicating that increased geographic distance between *P*. *calleryana* individuals does not necessarily mean increased genetic dissimilarity. The amplitude of the Mantel’s r scores in the correlogram ranged between about −0.10 and 0.15, indicating a relatively a low impact of spatial distancing on the population structure of the *P*. *calleryana* dataset.

Additionally, the phylogeographic signals within the *P*. *calleryana* dataset (North/South Groups) assessed using SPAGeDi indicated statistically similar results for F_ST_ and the mean permuted R_ST_ across all loci (*P*obs > exp = 0.48; data not shown). This suggested the absence of a phylogeographic signal within populations. To further evaluate the presence of a phylogeographic signal among populations, the slope test (b-log values) of pairwise R_ST_ were also evaluated. No evidence of phylogeographic signal was demonstrated among populations (*P*obs > exp = 0.95; data not shown).

#### Structure and Discriminant Analysis of Principal Components

**FIGURE 3:**
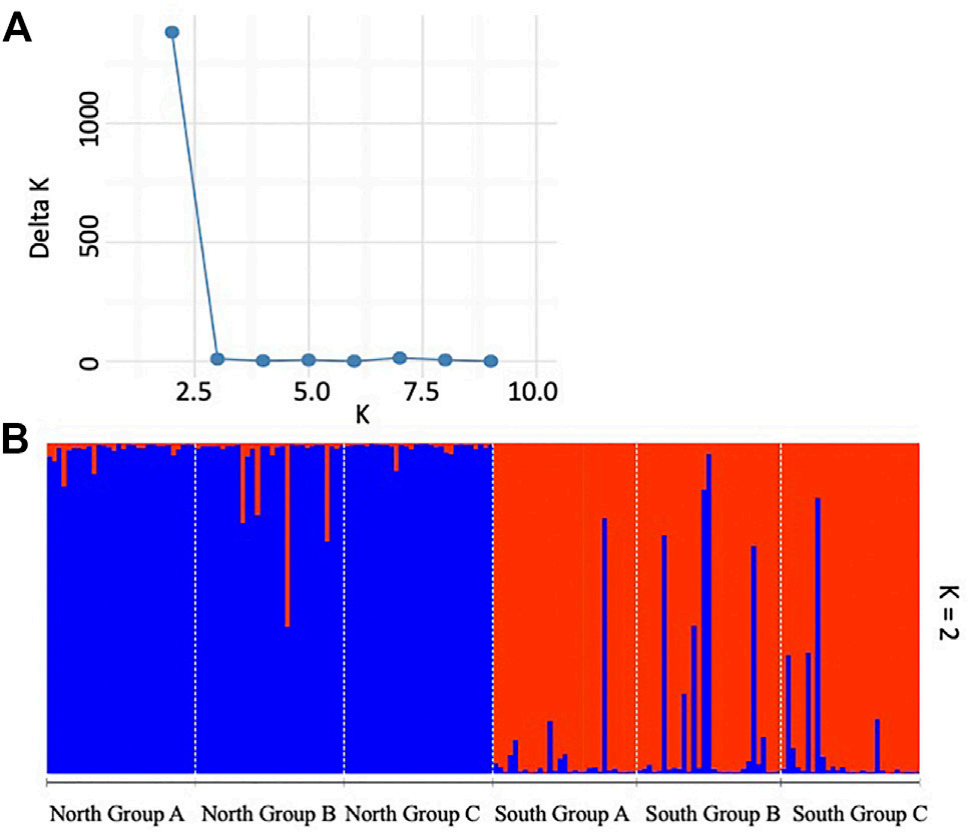
Bayesian clustering using Structure for the *Pyrus calleryana* dataset. Results were analyzed using **(A)** the Evanno method and visualized using **(B)** two (K = 2) inferred genetic clusters. An individual sample is represented by each vertical bar and an individual’s probability to belong to the identified cluster is represented by the blue versus red bar colors.

Bayesian clustering analysis using Structure indicated an optimum of ΔK = 2 suggesting the presence of two genetically distinct clusters among the studied subpopulations of *P*. *calleryana* (Figure 3). The result consisted of 2 genetic clusters comprising of 3 subpopulations from each North Group and South Group, respectively, with limited admixture between them.

The overall ObStruct’s R^2^ between the predefined populations and inferred clusters under K = 2 was 0.88 ± 2.28E-16 suggesting a strong divergence among the predefined major groups and between the Structure-derived genetic clusters within the dataset (Supplementary Table S2). Only negligible changes in R^2^ were evident when the predefined populations were sequentially removed. There was also no change in R^2^ when the inferred clusters were sequentially removed, suggesting no major contribution imparted by the inferred clusters to the population structure of the *P*. *calleryana* dataset. As such, the results of successive removal of populations/clusters imply that our *P*. *calleryana* populations might be a part of an even bigger community of *P*. *calleryana*.

**FIGURE 4:**
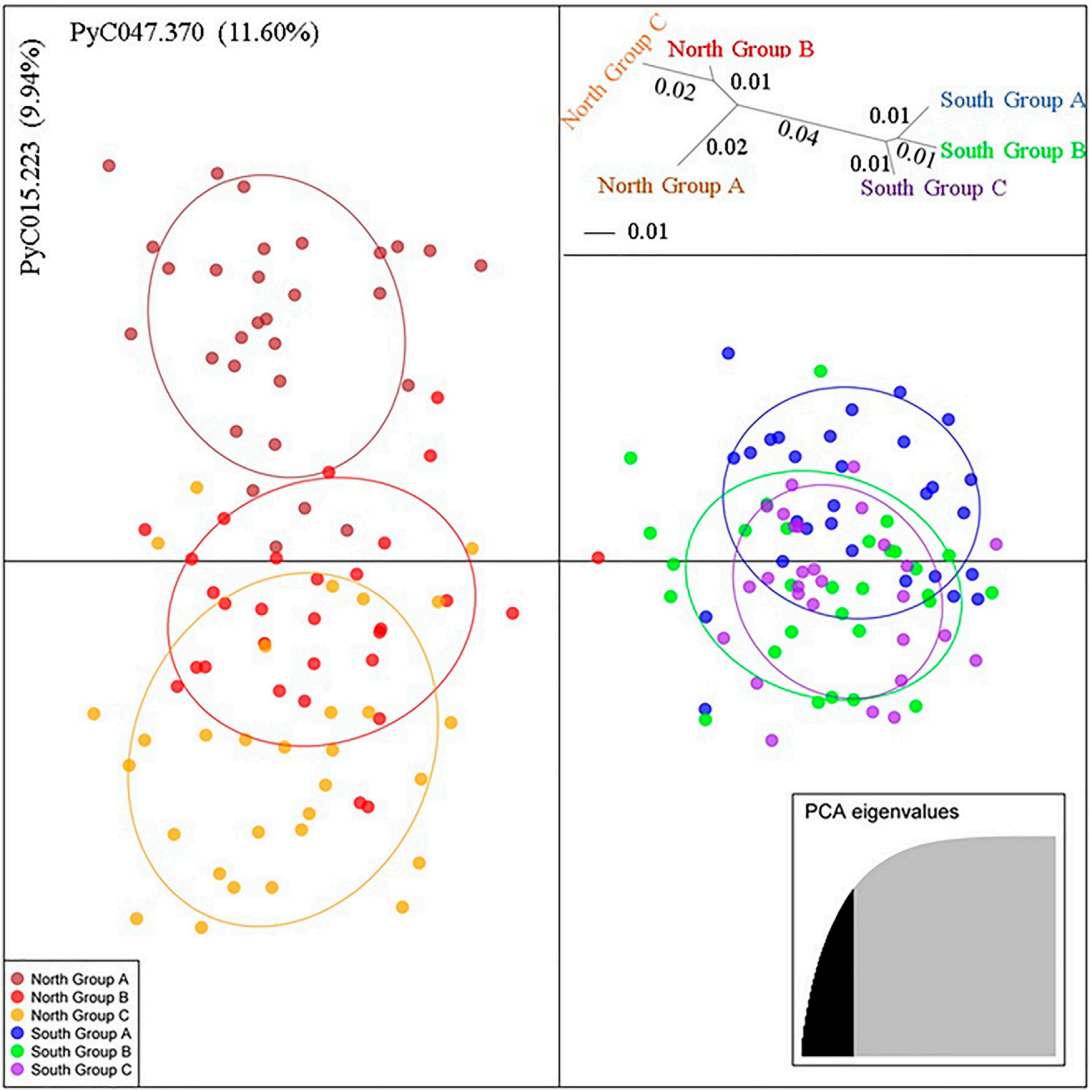
Discriminant Analysis of Principal Components (DAPC) of the *Pyrus calleryana* dataset. The alleles that explained the most of variance within the sampled populations (and their contributions) are indicated along *X* and *Y* axes. Each of the six colors represent six subpopulations (Brown: North Group A, Red: North Group B, Orange: North Group C, Blue: South Group A, Light Green: South Group B, and Purple: South group C), indicated in the legend (bottom-left). The genetic distance tree (insert top right) represents the unrooted neighbor-joining tree of pairwise genetic distances (Nei, 1978) among the sampled 176 *P*. *calleryana* individuals. This genetic distance for each split is followed by 70% or higher bootstrap, based on 1000 permutations of the distance matrix (distance/bootstrap). As shown in the bottom-right insert, 33 PCAs that saturated the cumulative variance at about 80% were selected, to visualize the dataset.

A multivariate analysis, DAPC, for the *P*. *calleryana* dataset showed a clustering pattern similar to that of Structure (Figure 4). The *P*. *calleryana* dataset was divided into two major clusters similar to their geographical location. This result was further supported by an unrooted neighbor-joining tree of pairwise genetic distances among the sampled *P*. *calleryana* individuals (Figure 4, insert top right).

### Population Demography

#### Bottleneck

Using the Wilcoxon test in Bottleneck, significant signals were found under the T.P.M. and S.M.M. mutation models that indicated presence of a possible bottleneck (heterozygosity deficiency) in both North and South Groups. The mode shift in the population size was analyzed using the information from all three basic tests (i.e., sign, standardized differences, and Wilcoxon sign-rank tests). In the cumulative mode shift test, a normal L-shaped distribution was detected in the North Group and South Group signifying no evidence of recent bottleneck events (Supplementary Table S3).

#### Approximate Bayesian Computation

**FIGURE 5:**
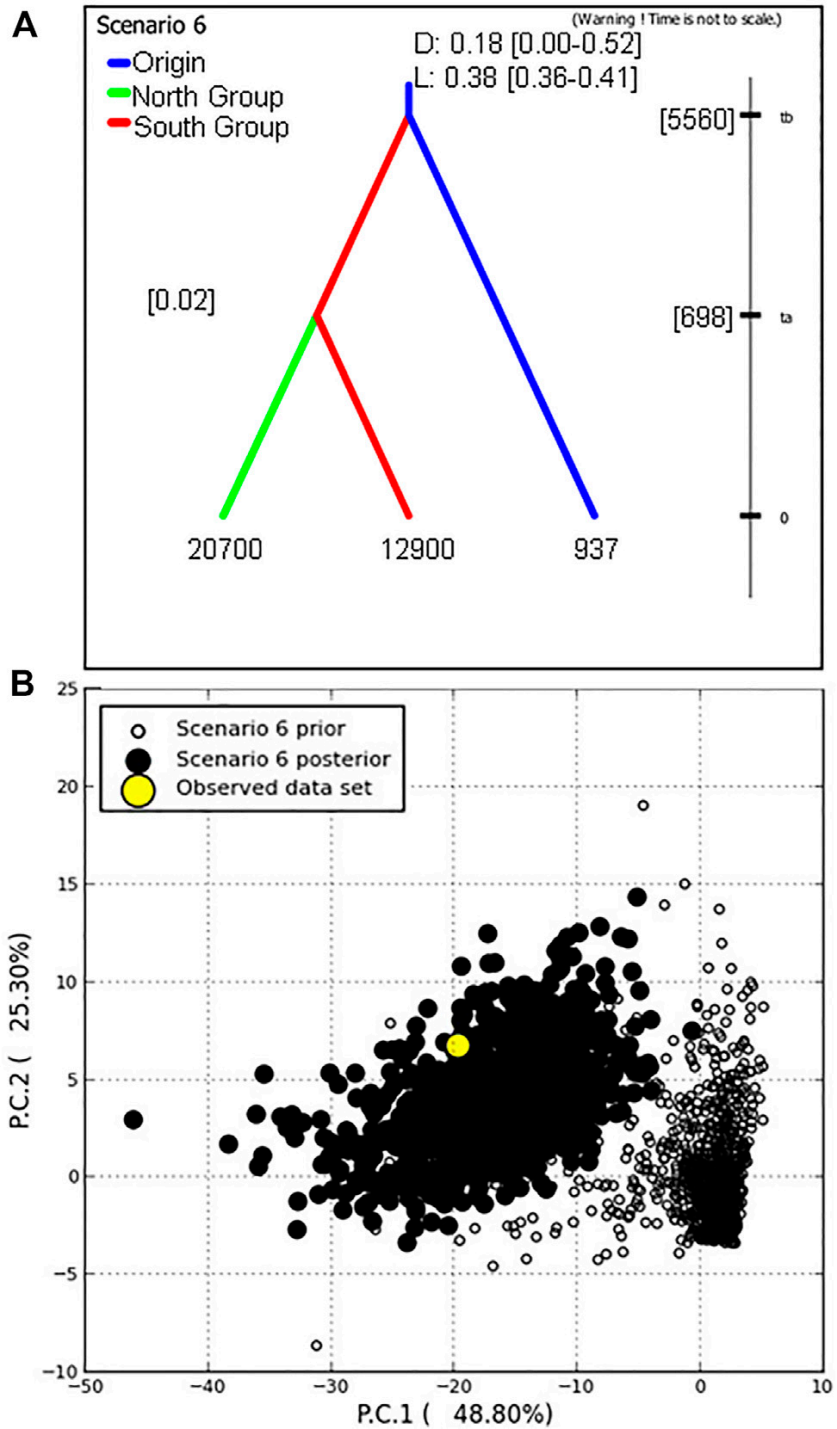
The best-supported scenario by DIYABC (Scenario 6) for the genotyped *Pyrus calleryana* dataset. **(A)** Scenario 6 had the highest support; here the Origin Group (relative effective population size of about 937 individuals) diverged the South Group (effective population size of about 12900 individuals) at about 5560 generations into the coalescent, from which the North Group diverged at about 698 generations into the coalescent (effective population size of about 20700 individuals). D and L indicate the relative support values derived from direct and logistic regression approaches, respectively, with their probability values of 95% confidence intervals given in square brackets ([]). “t” represents the time of occurrence of events expressed in generations. **(B)** Model-checking of the closest 1% simulated prior and posterior datasets was performed using two PCAs explaining the most variance in the summary statistics.

After the initial analysis parameters were established, six hypothetical evolutionary scenarios were devised from historical *P. calleryana* introduction to US and further evaluated. Of the six scenarios, we found the highest relative support for evolutionary scenario 6 (Figure 5, Supplementary Figure S4), followed by scenario 5 (Supplementary Figure S4). In scenario 6, we assumed the divergence of the South Group from the Origin Group, and at later time, the divergence of the North Group from the South Group (Figure 5A). In the competing scenario 5, “North Group” was derived from the “Origin Group,” whereas “South Group” in turn diverged from “North Group.” Scenario 6 was accepted as the most likely evolutionary scenario for the analyzed *P*. *calleryana* dataset as it had the highest relative posterior probability, highest relative support by logistic regression, and the range of 95% CI did not overlap with the CI ranges of other models. The related posterior parameter estimates under scenario 6 (Table 4) suggested the high mutation rate of 0.01 per locus per generation. In accordance with our assumptions, these data indicated that both “North Group” and “South Group” of *P*. *calleryana* evolved from the Origin Group. The relative mean absolute deviation for the *P*. *calleryana* dataset derived using prior and posterior distributions was 0.44 (95% coverage: 0.94) and 1.35 (95% coverage: 0.79), respectively (Supplementary Table S4). The confidence prior type I error in scenario 6 for the genotyped *P*. *calleryana* dataset using the direct and logistic approach was 0. 47 and 0.54, respectively, whereas the confidence prior type II error in scenario choice using the direct and logistic approach was 0.54 and 0.58, respectively (Supplementary Table S5).

**TABLE 4:** DIYABC analyses of trees genotyped in the *Pyrus calleryana* dataset for the evolutionary scenario 6.

Parameter	Mean	Median	Mode	q025	q050	q250	q750	q950	q975
N_Origin Group_ ^a^	974	937	879	352	433	719	1180	1630	1810
N_North Group_	24900	20700	15000	5900	7280	13900	31100	59600	68700
N_South Group_	2560	1290	876	322	420	836	2150	7660	14000
t1	989	698	320	122	171	404	1230	2810	3710
t2	5640	5560	4690	1510	1930	3800	7540	9450	9720
µmic1	0.013	0.012	0.010	0.003	0.004	0.008	0.017	0.028	0.034

a N_X_: effective size of the given population; t_X_: estimated time since split (generations); µmic_1_: overall mutation rate (mutations per locus per generation). The generated individual numbers are all relative and estimated calculations which derives from varying population sizes with varying priors.

## Discussion

Despite its ubiquitous presence across much of the eastern US, we know little about the evolutionary history of *P*. *calleryana* in eastern North America. This study shows that naturalized populations of *P*. *calleryana* have recently diverged to become invasive within the United States. Further, our data indicates an extremely high evolutionary potential of *P*. *calleryana* due to high genetic diversity. The species thus presents a great threat to ecosystem sustainability and biodiversity of native plant species and a high likelihood of continued spread of open-pollinated *P*. *calleryana* escapees. We also identified several features underlying the documented intraspecific hybridization in this species (Whitehouse et al., 1963; USDA, 1981; Culley and Hardiman, 2009; Dunn, 2018). The high mutation rate detected here can explain the excess of alleles per population over the expected number of effective alleles. Structure and DIYABC analyses also showed strong differentiation between North and South Groups, possibly deriving from regional preferences or availability of different cultivars that are transplanted into either region and that give rise to the escape populations (Culley and Hardiman, 2009; Dunn, 2018). These findings can explain how contemporary *P*. *calleryana* individuals could evolve in about 120 years from a relatively small number of introduced individuals into an invasive population that has become established on a continental scale.

Our study showed high *P*. *calleryana* genetic diversity, similar to genetic diversity reported for a related species, *Malus orientalis* Uglitzk. in Iran using nine SSRs (H_e_ = 0.76; Farrokhi et al., 2011) and somewhat higher than that reported for *P*. *calleryana* in China using 14 nuclear SSRs (H_e_ = 0.64; Liu et al., 2012). The genetic diversity statistics for *P*. *calleryana* were also higher compared to other invasive plant species, such as *Albizia lebbeck* (L.) Benth. and *Pueraria lobata* Willd. (Pappert et al., 2000; Dunphy and Hamrick, 2005). Our results support the hypothesis that escaped *P*. *calleryana* trees have high genetic diversity, which could be the result of high gene flow, whereas high genetic differentiation among populations may stem from multiple introductions of genetically different rootstocks and cultivars into landscapes across time and their intraspecific hybridization (Culley and Hardiman, 2009). We observed extremely high evolutionary potential underscored by high mutation rates and high genetic diversity, likely aided by widespread distribution of the species through various dispersal mechanisms via vertebrates, insects, and humans (Hamrick et al., 1992; Ellstrand and Schierenbeck, 2000; Sexton et al., 2002; Dlugosch and Parker, 2008; Culley et al., 2011). Multiple dispersal mechanisms increase the chances for survival of the evolutionary successful allele combinations and thus impact the species demographics, in addition to various consequences for gene flow (Culley and Hardiman, 2009; Dunn, 2018). In the case of *P. calleryana*, this is further enhanced by virtual absence of major pathogens or pests (Dirr, 2009; Lalk et al., 2021; Farr and Rossman, 2022; Hartshorn et al., 2022) and general hardiness (Randhawa, 1949; Whitehouse et al., 1963). It is plausible that the high portion of within-individuals diversity in 3-tier AMOVA versus 2-tier AMOVA, which reflects the local gene dispersal, contributes to the genetic diversity within North Group and South Group (Dunn, 2018). Occasional migrants across the Appalachian Mountains that represent the intuitive geographic barrier, further confirmed by the results of Structure and DAPC, may have contributed to the overall genetic diversity of the escaped *P. calleryana*. Those cross-regional migrants could be due to long-distance seed dispersal (Culley and Hardiman, 2009) or human-based transplantation. Other possible gene flow barriers may stem from the local environmental characteristics, as *P. calleryana* prefers certain soil types, moisture levels, and nutrients compositions (Randhawa, 1949). These factors provide potential elements that would be expected to influence the appearance of new local populations as has been suggested by other studies (Dunn, 2018) and are currently under investigation. Our study also supports the observation that outcrossing species tend to have higher levels of within-population genetic diversity and lower levels of among-population genetic diversity (Hamrick and Godt, 1996).

Isolation by distance indicated a positive correlation between genetic and geographic distance, implicating geographic distance as one of the factors in determining the genetic structure of the *P*. *calleryana* dataset, albeit with a low effect. This positive correlation implies an existence of local barriers to the gene dispersal at regional or subpopulation level, in addition to the major geographical barrier caused by the regional differentiation. Such barriers are more likely to influence *P. calleryana* short-distance cross-pollination by several generalist pollinators than the long-distance seed dispersal by birds (Culley and Hardiman, 2009). Compared to our study, a higher positive correlation between genetic and geographic distances was obtained for collection of wild *P*. *calleryana* in China, where most *P*. *calleryana* trees predominantly grow in fragmented and isolated clusters (Liu et al., 2012). The present status of *P*. *calleryana* across its native ranges in China and Japan indicated that native populations are fragmented and nearing extinction due to urbanization (Liu et al., 2012; Kato et al., 2013), whereas it is widely regarded as invasive in the United States (Culley et al., 2011; Coyle et al., 2021; Lalk et al., 2021). Furthermore, no significant differences between the permuted values of R_ST_ and F_ST_ were found, indicating the absence of phylogeographic patterns within our *P*. *calleryana* dataset, and the mutation rate contributing *en par* with the vast migration rate to the species genetic variability (Hardy and Vekemans, 2015; Nowicki et al., 2020).

Only the North Group presented two private alleles. The low number of private alleles in *P*. *calleryana* population is corroborated by the high gene flow among *P*. *calleryana* individuals. This may indicate presence of the compatible *P*. *calleryana* specimens in nearby locations cross-pollinating with each other, and an intensive seed dispersal to distant and nearby locations by animals. The genetic diversity of the North Group was not significantly higher than that of the South Group, and other genetic diversity indices were also comparable for both groups. High gene flow estimates among escaped *P*. *calleryana* populations were consistent with our previous study of the native Asian collection and US cultivars of *P. calleryana* (Sapkota et al., 2021), as well as in studies of other invasive species such as *A. lebbeck* (Dunphy and Hamrick, 2005) and *Fallopia* species (Gaskin et al., 2014). A high rate of gene flow helps in the allele exchange among populations and ensures abundant fruit crops in self-incompatible species contributing the seeds for population growth and colonization of new areas (Dunphy and Hamrick, 2005). Compared to other trees, *P*. *calleryana* fruits stay on the trees longer, thereby becoming an emergency food for birds and other vertebrates during winter when other food sources are scarce (Culley and Hardiman, 2009; Culley, 2017). Persistent retention of fruit may ultimately facilitate the dispersal of seeds to distant areas and enhance the capability of open-pollinated *P*. *calleryana* to become highly successful as an invasive species, as documented by a very minor cross-regional admixture in our Structure results. *Pyrus calleryana* trees are visited by various pollinators such as honeybees and frugivorous animals leading to short- and long-distance dispersal of both pollen and seed (Culley and Hardiman, 2007; Culley and Hardiman, 2009; Liu et al., 2012). There is also extensive human-mediated dispersal of *P*. *calleryana* trees via selection, propagation, and transportation.

A high level of genetic differentiation was reported among populations as observed in other studies conducted on *P*. *calleryana* (Liu et al., 2012; Sapkota et al., 2021), as well as deciduous flowering tree species including *Cornus florida* and *C*. *kousa* (Nowicki et al., 2020) that have maintained high levels of genetic diversity. *Pyrus calleryana* has been able to maintain a high level of genetic differentiation despite the high gene flow among *P*. *calleryana* individuals within populations and the founder effect evident from historical data, heterozygosity deficiency as per Bottleneck, and DIYABC inferences. Maintenance of high genetic diversity could result from various dispersal mechanisms of *P*. *calleryana* trees and geographic barriers imposed between North Group and South Groups both by distance and the Appalachian Mountain range. There was a low value of standardized index of association ( $\bar{r}$ _d_) for our dataset, yet a positive inbreeding coefficient (F_IS_) was found in contrast to *P*. *calleryana* biology. Callery pear is an outcrossing species (Culley and Hardiman, 2009) and would be expected to undergo random mating; as such, the finding of positive F_IS_ could signify a founder effect and the alleles deriving from a limited pool of ancestors. Such positive F_IS_ for our dataset could be also the result of human interference, for example with regard to selection, propagation, multiple local introductions, and intentional transportation of *P*. *calleryana* leading to the escape of the species. Likewise, insect pollination limiting the long-distance pollen flow and the historically-documented founder effect are expected to influence positive F_IS_. The positive F_IS_ we observed is consistent with results reported from other studies conducted within *P*. *calleryana*’s native range (Liu et al., 2012; Sapkota et al., 2021).

The DIYABC inferences add information about how *P. calleryana* could overcome the limitations imposed by the self-incompatibility and the founder effect. We observed a very high mutation rate for *P. calleryana*, compared with other species occupying similar ecological niches (Nowicki et al., 2020; Ony et al., 2020; Ony et al., 2021). This feature, in addition to high gene flow, could contribute to the observed heterozygosity and to increasing the population differentiation under the presence of barriers. But, the split times observed defy the documented history of the species in the United States (Whitehouse et al., 1963; Vincent, 2005), exceeding the number of generations about 50-fold. This implies extremely high levels of intraspecific hybridization and thus increases alarm about the ongoing *P. calleryana* invasion.

Information from this study sheds light on the population dynamics of this invasive species with an outlook to its continued adaptation and spread. Furthermore, our study provides a great prospect for future research on the invasive *P*. *calleryana*. Our data could be enhanced by a broad-scale *P*. *calleryana* genomics study that evaluates trees from across a wider geographic range. This genomic approach could help us understand other aspects of *P*. *calleryana* not covered in microsatellite-based studies, such as investigating the molecular mechanisms underlying reproductive incompatibility, and genomic features enabling the evolutionary success of *P*. *calleryana* despite observed high inbreeding and self-incompatibility. Results from that work are expected to help us compare the genomic characteristics of *P*. *calleryana* to our present findings and will help us better understand the invasive character of the species at its molecular background, potentially informing strategies for effective management.

## Data Availability

The datasets presented in this study can be found in online repositories. The names of the repository/repositories and accession number(s) can be found in the article/Supplementary Material.
