# Supplementary material for: Microsatellite Loci Reveal High Genetic Diversity, Mutation, and Migration Rates as Invasion Drivers of Callery Pear (Pyrus calleryana) in the Southeastern United States
Source: Front Genet. 2022 Apr 5;13:861398. doi: 10.3389/fgene.2022.861398 (PMC9037086; doi:10.3389/fgene.2022.861398)
Supplement: Supplementary file 1 [file DataSheet1.docx]

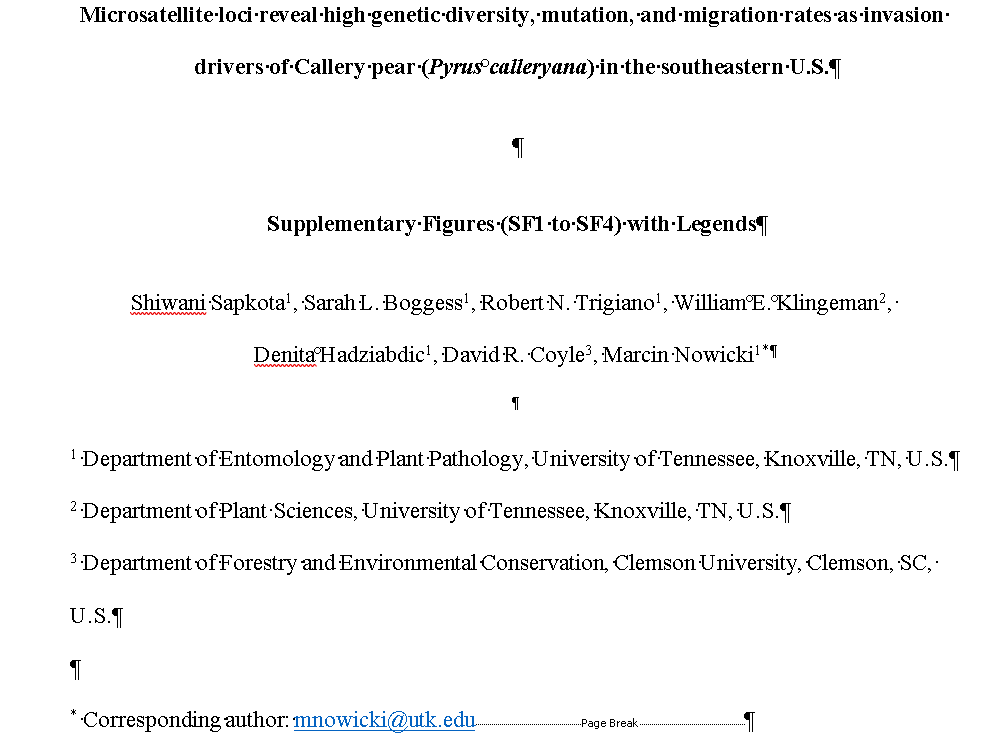


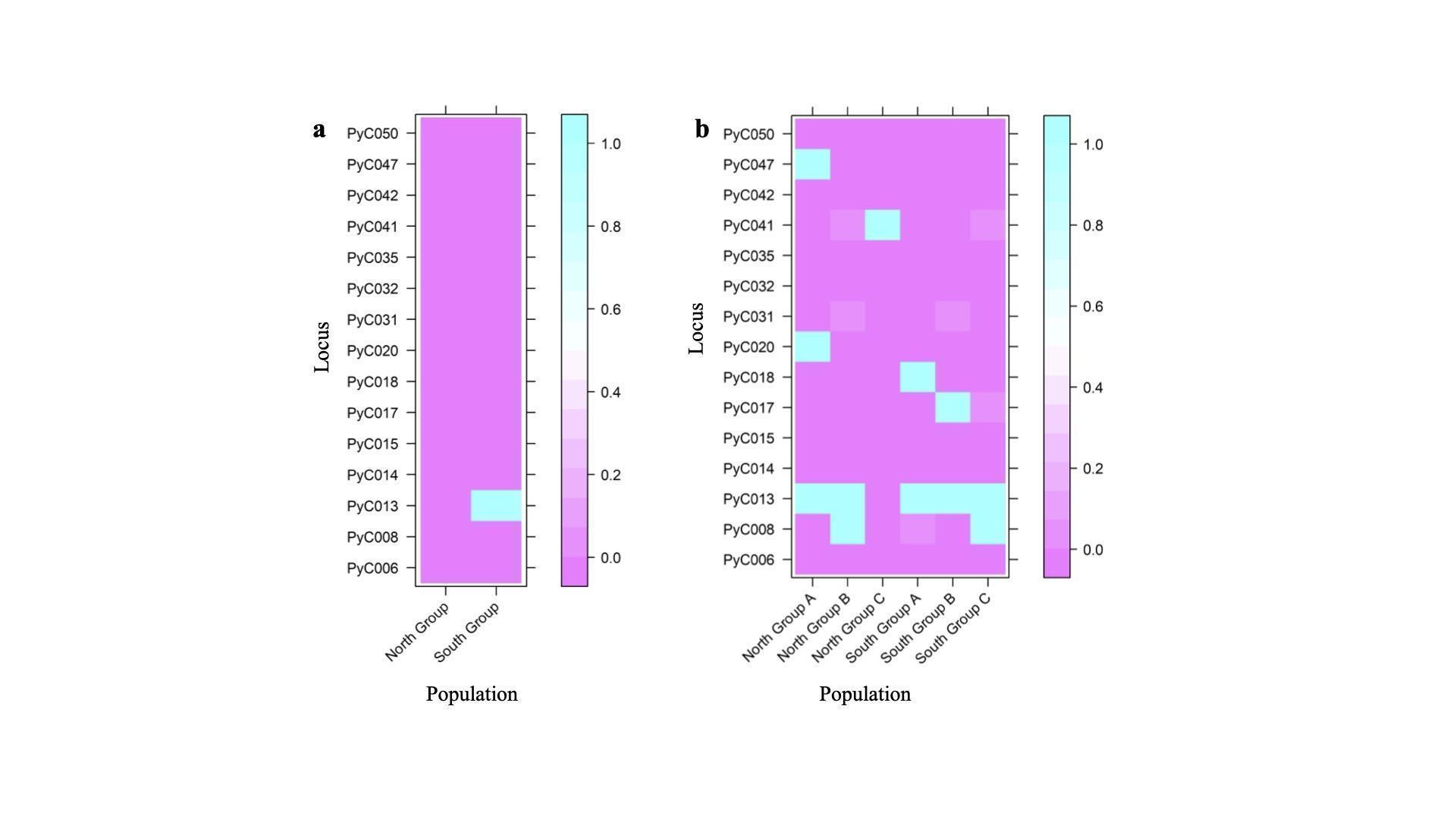


**Supplementary Figure 1.** Hardy-Weinberg Equilibrium (HWE) for six subpopulations and loci of trees comprising the *Pyrus* *calleryana* dataset. (**a**) HWE for North and South Groups dataset subdivision, and (**b**) HWE for six subpopulations dataset subdivision. Rows represent the loci, and columns represent the sample populations used for the study. The probability of the given loci following HWE is shown in the legend. The deep-pink color represents the loci not in HWE at *P* ≤ 0.05.


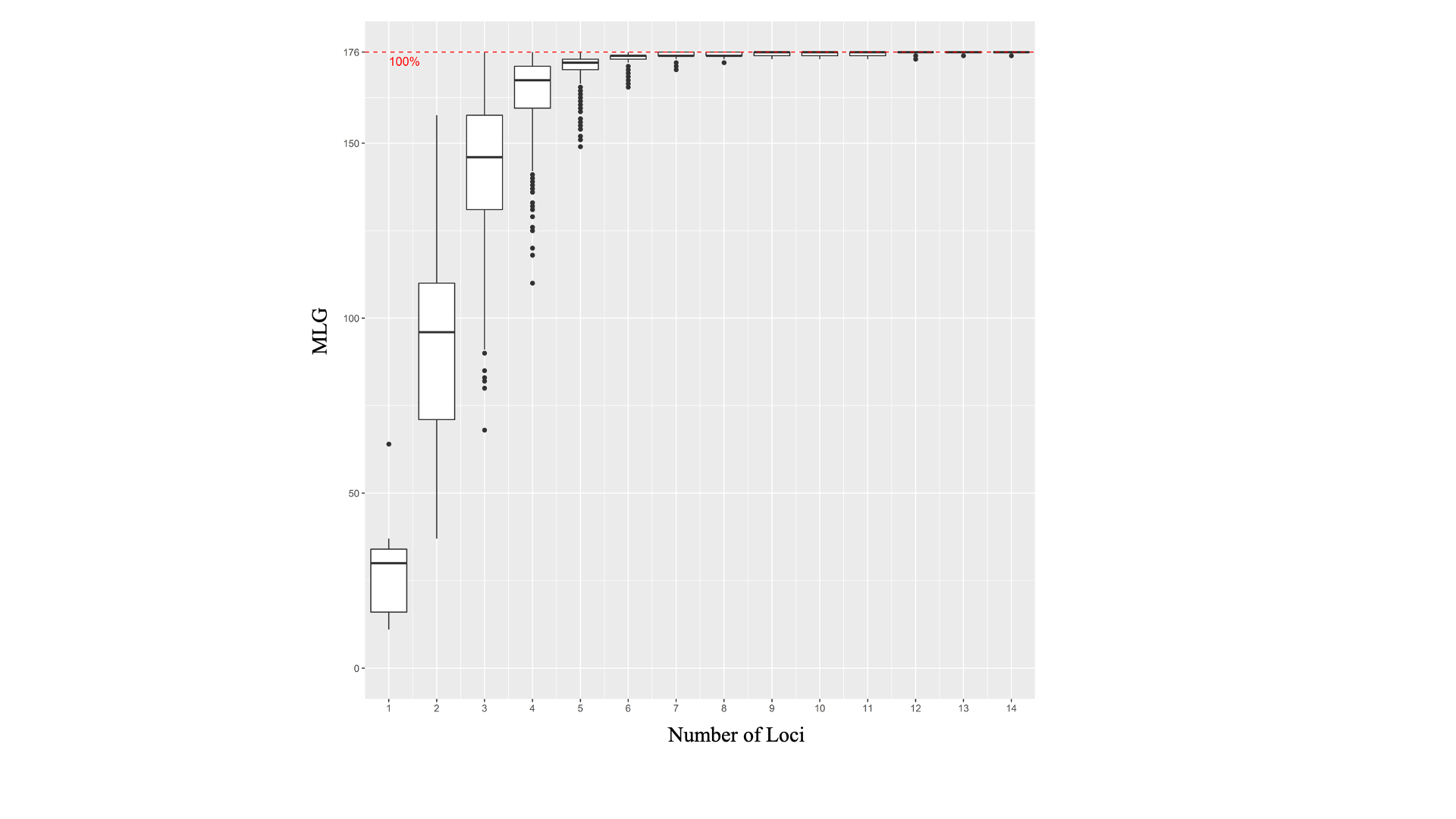


**Supplementary Figure 2.** Genotype Accumulation Curve (GAC) for trees comprising the *Pyrus* *calleryana* dataset. The X-axis represents the number of loci sampled and the Y-axis represents the number of multi-locus genotype (MLG) detected.


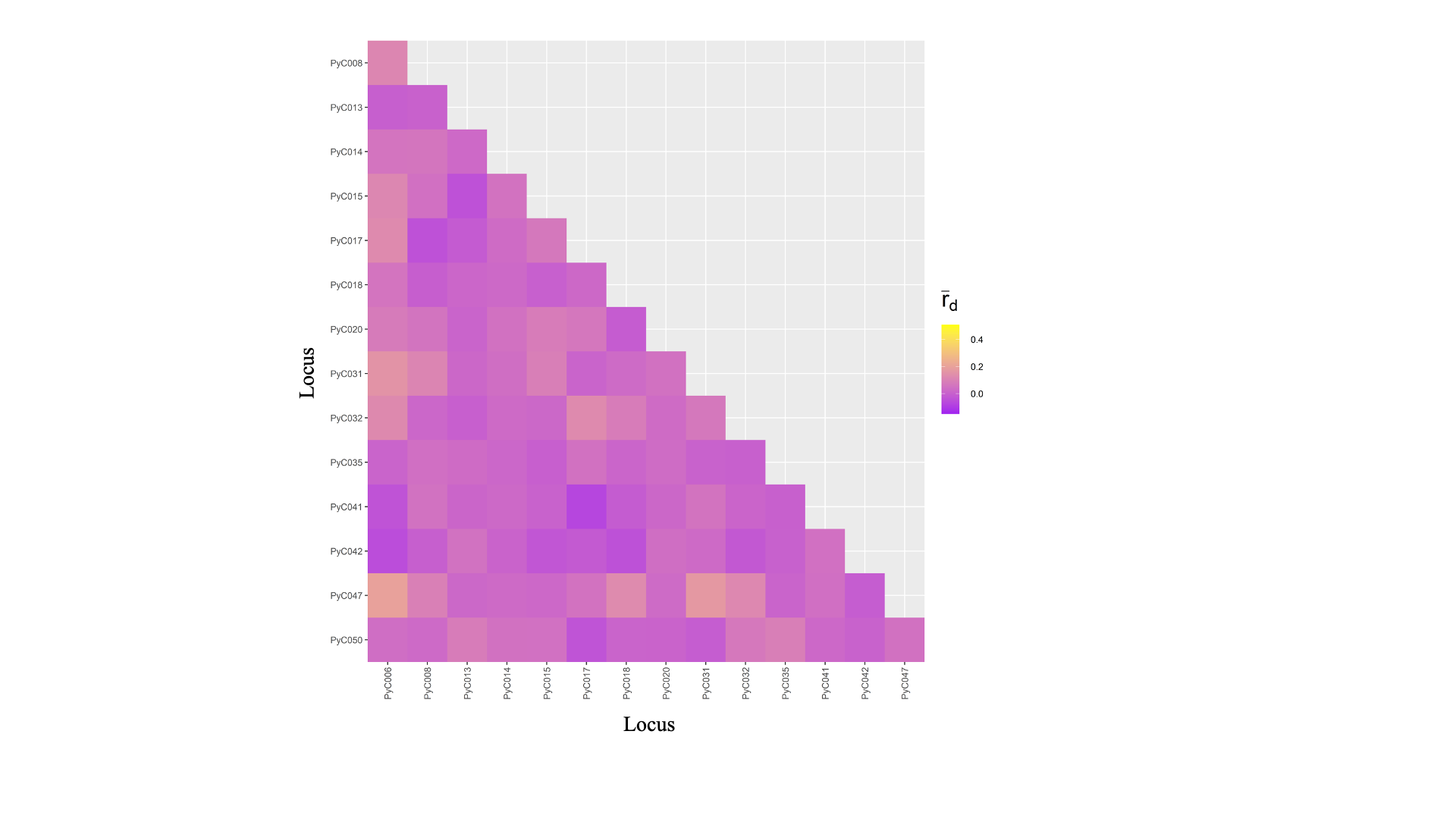


**Supplementary Figure 3.** Standardized pairwise Linkage Disequilibrium ($\overline{r}$_d_) among the studied 15 loci used to genotype the tree samples comprising the *Pyrus* *calleryana* dataset. The linkage strength between pairs of loci is represented by hues explained in the legend.


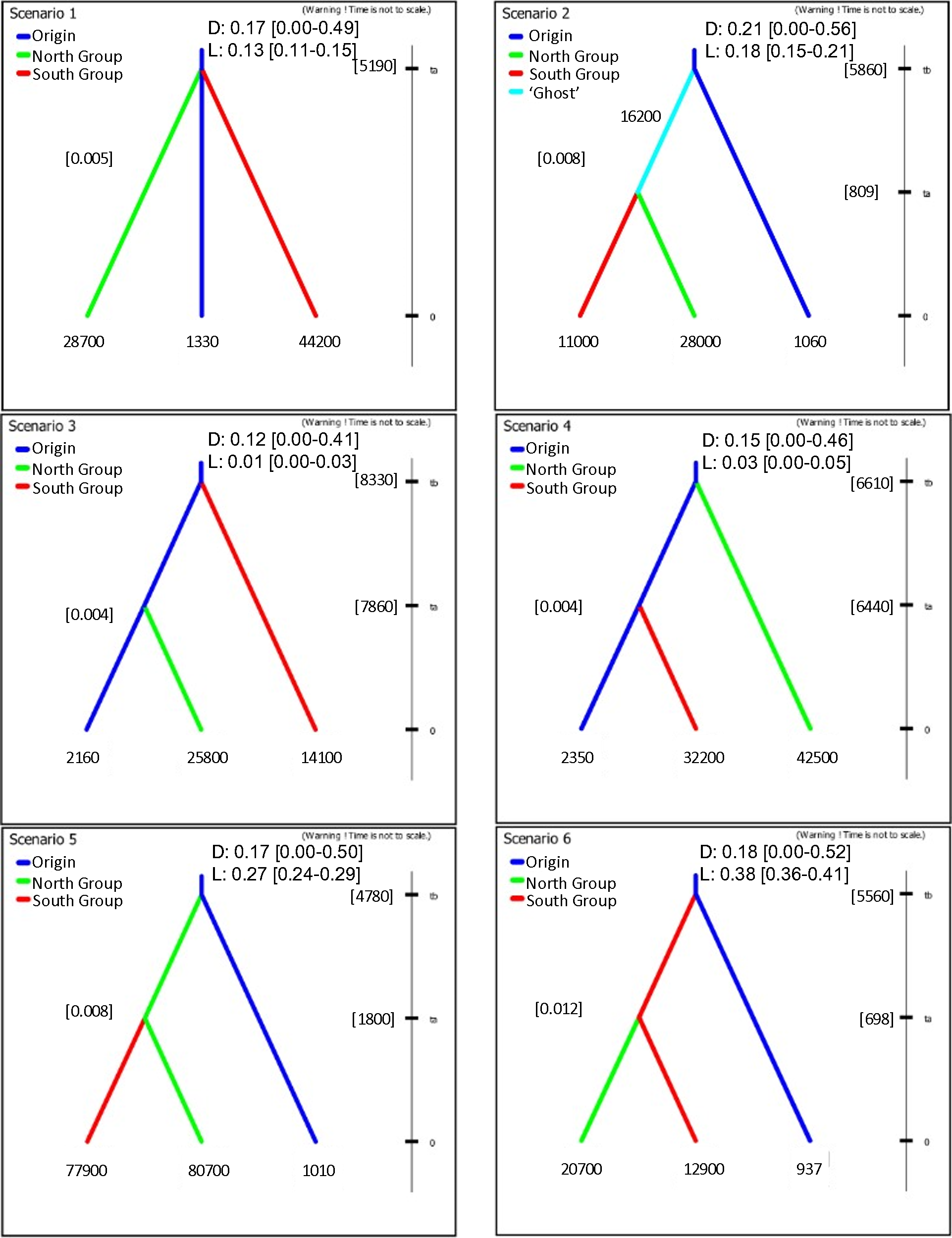


**Supplementary Figure 4.** The scenarios compared for the evolutionary history of the *Pyrus* *calleryana* using DIYABC. In total, 6 hypothetical evolutionary scenarios were considered and tested using the North Group, South Group, Origin Group, and ‘Ghost’ population. In the figure, the numbers (construct number generated by DIYABC for each scenario) given below each population name represent the median of effective population size for each population, respectively. For each of the tested scenarios, D and L indicate the relative support values derived from direct and logistic regression approaches, respectively, with their probability values of 95% confidence intervals given in square brackets ([]). ‘t’ represents the time of occurrence of events in terms of generations.
